# Impaired Regeneration in Dystrophic Muscle—New Target for Therapy

**DOI:** 10.3389/fnmol.2020.00069

**Published:** 2020-05-25

**Authors:** Nurit Yanay, Malcolm Rabie, Yoram Nevo

**Affiliations:** ^1^Felsenstein Medical Research Center (FMRC), Tel-Aviv University, Tel-Aviv, Israel; ^2^Institute of Neurology, Schneider Children’s Medical Center, Tel-Aviv University, Tel-Aviv, Israel

**Keywords:** LAMA2-CMD, laminin-211, next-generation sequencing, satellite cells, *dy*^2J^/*dy*^2J^ mouse model, muscular dystrophy

## Abstract

Muscle stem cells (MuSCs), known as satellite cells (SCs) have an incredible ability to regenerate, which enables the maintenance and growth of muscle tissue. In response to damaging stimuli, SCs are activated, proliferate, differentiate, and fuse to repair or generate a new muscle fiber. However, dystrophic muscles are characterized by poor muscle regeneration along with chronic inflammation and fibrosis. Indications for SC involvement in muscular dystrophy pathologies are accumulating, but their contribution to muscle pathophysiology is not precisely understood. In congenital muscular dystrophy type 1A (LAMA2-CMD), mutations in *Lama2* gene cause either complete or partial absence in laminin-211 protein. Laminin-211 functions as a link between muscle extracellular matrix (ECM) and two adhesion systems in the sarcolemma; one is the well-known dystrophin–glycoprotein complex (DGC), and the second is the integrin complex. Because of its protein interactions and location, laminin-211 has a crucial role in muscle function and survival by maintaining sarcolemma integrity. In addition, laminin-211 is expressed in SCs and suggested to have a role in SC proliferation and differentiation. Downstream to the primary defect in laminin-211, several secondary genes and pathways accelerate disease mechanism, while at the same time there are unsuccessful attempts to regenerate as compensation for the dystrophic process. Lately, next-generation sequencing platforms have advanced our knowledge about the secondary events occurring in various diseases, elucidate the pathophysiology, and characterize new essential targets for development of new treatment strategies. This review will mainly focus on SC contribution to impaired regeneration in muscular dystrophies and specifically new findings suggesting SC involvement in LAMA2-CMD pathology.

## Introduction

Skeletal muscle tissue is the most abundant tissue in the body. It is dynamic and in its healthy state adaptable to changes, such as exercise or injury. Skeletal muscle has the ability to fully regenerate in response to injury and to increase cell number or size accordingly. Muscle adaptive behavior is maintained by muscle stem cells (MuSCs), also known as satellite cells (SCs), located in a niche in close proximity to the muscle fiber. In response to either intrinsic or microenvironment (extrinsic) signaling, SCs are activated, proliferate, and fuse in a very controlled manner to repair or create new muscle fiber.

Muscular dystrophies are characterized by progressive skeletal muscle weakness and atrophy. The clinical deterioration is caused by substitution of muscle by fibrotic and fatty nonfunctional tissues. With new advanced diagnostic methods, accumulating data are emerging regarding decreased SC function and number, leading to impaired regeneration as a contributory mechanism to the pathology in muscular dystrophy. In this review, we mainly focus on impaired regeneration and SC involvement in the pathology of muscular dystrophies and the new findings in LAMA2-CMD.

## Skeletal Muscle Regeneration

**Figure 1:**
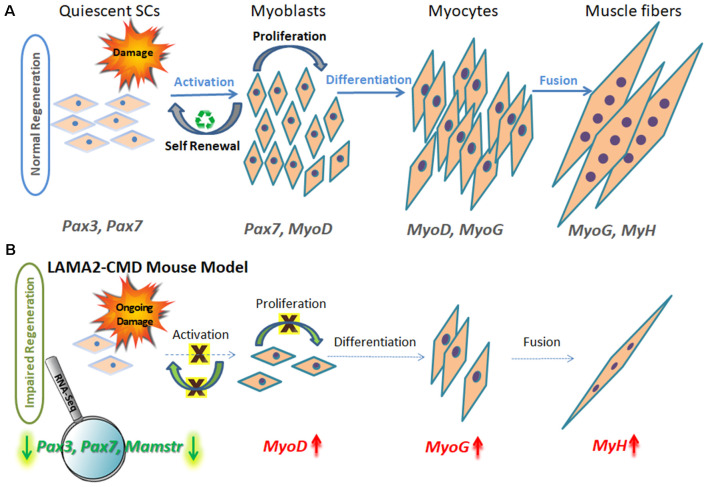
Schematic model of muscle regeneration process in normal and LAMA2-CMD mouse model. **(A)** In healthy muscle, satellite cells (SCs) are in a quiescence state as a default, located in a specialized niche, between the sarcolemma and basal lamina. Quiescent SCs express Pax7, and a subpopulation expresses also Pax3 transcription factors; however, MyoD is not expressed at this stage. Upon injury, SCs are activated and undergo proliferation. SCs can perform asymmetric or symmetric divisions. Asymmetric division allows self-renewal and maintenance of SC pool, and symmetric division allows myogenesis and generating myoblasts. Following proliferation stage, myoblasts express MyoD transcription factor and exit the cell cycle to promote differentiation. Upon differentiation, myoblasts differentiate into elongated myocytes expressing myogenin (MyoG). Myocytes can fuse forming myotubes, which become myofibers, the contractile unit of muscle, and express developmental myosin-heavy chain marker (MyH). **(B)** In dystrophic muscle, SCs have limited ability to compensate for muscle damage. In LAMA2-CMD *dy^2J^/dy^2J^* mouse model, RNA-Seq data indicated little or even absent SC population, due to significant downregulation of Pax7 and Pax3 genes in quadriceps muscle of 8-week-old mice (Yanay et al., 2019). Absence of those transcription factors disable proper proliferation and self-renewal; thus, the entire regeneration process is impaired. MyoD and MyoG upregulation is postulated to be an attempt to compensate for the unbalanced process and the reduced repair of the damage.

Adult healthy skeletal muscles have an excellent regeneration capacity to undergo constant repair and create new muscle fibers owing to MuSCs, also known as SCs. SCs are located in a niche between the myofiber sarcolemma and basement membrane, near the vasculature, and thus can act very rapidly, migrating and proliferating upon muscle injury (Mauro, 1961), and are the primary source of muscle regenerative cells (Ciciliot and Schiaffino, 2010; Almeida et al., 2016). Many other mononuclear cells, such as bone marrow stem cells, mesenchymal stem cells, and pericytes accompanying the muscle microenvironment, also have a role in muscle homeostasis and repair (Ferrari et al., 1998; Corti et al., 2002; Lee et al., 2005; Muskiewicz et al., 2005; Dellavalle et al., 2007; Liu et al., 2007; Negroni et al., 2009; de la Garza-Rodea et al., 2011). Following muscle injury, necrosis of damaged myofibers is followed by inflammatory responses, including recruitment of neutrophils and macrophages, which secrete inflammatory cytokines activating the quiescent SC mononuclear population in order to regenerate muscle (Otis et al., 2014). Muscle regeneration is a controlled finely tuned process, very similar to muscle formation during embryonic development (Allbrook, 1981), and can be subdivided into three main stages: proliferation, differentiation, and fusion (Randolph and Pavlath, 2015; Chal and Pourquié, 2017; Yanay et al., 2019), as can be seen in Figure 1A.

Each stage of muscle regeneration can be classified by a variety of molecular markers. The most familiar marker of all muscle SC state is paired box transcription factor 7 (Pax7), being essential for SC function during postnatal growth both in regeneration of skeletal muscle and maintaining a renewable SC pool (Seale et al., 2000; Olguin and Olwin, 2004; Zammit et al., 2004; Lepper et al., 2009; von Maltzahn et al., 2013). Subsets of SCs express premyoblast marker paired box transcription factor 3 (Pax3), important in the initial steps of muscle formation (Relaix et al., 2005, 2006); however, Pax3’s role in adult human muscle still remains to be established. The population of Pax3 and Pax7 double-positive stem cells expression is observed in the preliminary stage throughout embryonic and fetal development, as well as later in adult skeletal muscle SCs. Pax3 and Pax7 stimulate gene expression promoting proliferation and asymmetric divisions (known as self-renewal) and repress genes driving differentiation (Ben-Yair and Kalcheim, 2005; Kassar-Duchossoy et al., 2005; Relaix et al., 2005, 2006).

Upon SC activation, proliferating myoblasts coexpress Pax7 and MyoD transcription factors. From this stage, the differentiation process begins and is characterized by MyoD and MyoG expression.

Proliferating myoblasts either down-regulate Pax7 to differentiate or down-regulate MyoD to undergo self-renewal process, which maintains the SC pool. Maintaining the balance between myoblast proliferation and differentiation is crucial for the appropriate muscle regeneration process (Yablonka-Reuveni, 2011).

Under dystrophic conditions, muscle is persistently injured and degenerates (Otis et al., 2014) in a way that muscle necrosis surpasses regenerative capacity, and muscle repair cannot effectively compensate for damage. The muscle undergoes replacement by fibrotic tissue and fat leading to severe loss of muscle mass and function. Recently, there are cumulative indications of SC involvement in different muscular dystrophies in which SCs apparently fail to repair muscle damage efficiently (Logan et al., 2011; Ross et al., 2012; Urciuolo et al., 2013; Di Gioia et al., 2017).

## The Contribution of Satellite Cell Regeneration Failure to Muscular Dystrophy Pathophysiology

Intrinsic and extrinsic defects may occur at each stage of this complex multipart muscle regeneration process. Several theories for the limited regenerative capacity of SCs have been proposed by Randolph and Pavlath (2015) and the 240th ENMC workshop on “The involvement of skeletal MuSCs in the pathology of muscular dystrophies” (Morgan et al., 2019). These theories can be divided into impairment of intrinsic and extrinsic factors:

**Intrinsic Impairment in SCs**

1. Reduction in SC pool. Defects in self-renewal, SC exhaustion, or senescence eventually lead to reduction in SC number. Self-renewal defects causing ineffective generation of SCs were demonstrated in sarcoglycanopathy LGMD2C and 2F patients (Higuchi et al., 1999), Col6A1 knockout mice (Urciuolo et al., 2013; Gattazzo et al., 2014a), Sepn1^−/−^ murine model, and in selenoprotein-related myopathy (SEPN1-RM) patient muscle biopsies (Sacco et al., 2010; Castets et al., 2011). SC exhaustion caused by constant activation from ongoing cycles of degeneration and regeneration was demonstrated in *mdx* mouse model of Duchenne muscular dystrophy (DMD) and myoblasts isolated from aging DMD patients (Mouly et al., 2005; Sacco et al., 2010; Jiang et al., 2014). Lately self-renewal defect in SCs, in addition to exhaustion, was also observed in *mdx* mice indicating asymmetric division anomalies (Dumont et al., 2015). Senescence (premature aging) was demonstrated in both *in vitro* and *in vivo* models of LGMD2H mouse SCs (Kudryashova et al., 2012; Mokhonova et al., 2015) and myoblasts of human myotonic dystrophy type 1 (DM1) and 2 (DM2; Bigot et al., 2009; Beffy et al., 2010; Renna et al., 2014).
2. Defects in myoblast proliferation. Decreased proliferation of SCs has been observed in oculopharyngeal muscular dystrophy (OPMD) patients’ myoblasts (Périé et al., 2006), Emery–Dreifuss muscular dystrophy (EDMD) patient’s muscle tissue sections and cultured myoblasts (Meinke et al., 2015), muscle biopsies of a DM1 patient (Thornell et al., 2009), and in myoblasts of murine model of LGMD2O (Miyagoe-Suzuki et al., 2009). In patients and mouse model of Pompe disease, insufficient SC activation was noted, although their function and number seemed normal (Schaaf et al., 2018). Additionally, impaired SC transition from proliferation to differentiation has been demonstrated in biopsies from LGMD2A patients (Rosales et al., 2013).
3. Defect or delay in the differentiation stage was demonstrated for facioscapulohumeral muscular dystrophy (FSHD) in DUX4c-expressing C2C12 myoblasts (Bosnakovski et al., 2008). Defects in myoblast differentiation were also demonstrated in EDMD primary muscle and patient-derived myoblast cultures (Frock et al., 2006) and in C2C12 cells expressing mutated lamin A, representing the cellular model of AD EDMD (Favreau et al., 2004).
4. Defect or delay in the fusion stage was reported in FRG1 mouse model for FSHD primary myoblasts (Feeney et al., 2015) and limb-girdle muscular dystrophy type 2L (LGMD2L) *Ano5*^−/−^ knockout mouse model (Whitlock et al., 2018).

**Extrinsic Impairment in SC**

1. Hostile microenvironment of dystrophic muscle may not be permissive for continued SC regeneration (Boldrin et al., 2012). Altered composition of the extracellular matrix (ECM), chronic inflammation (Wanschitz et al., 2013), defective autophagy (Tang and Rando, 2014), and fibrosis lead to a defect in SC niche and to cell senescence, as demonstrated in mouse models of dystroglycanopathy (Ross et al., 2012).
2. Alterations in signaling pathways as a result of the dystrophy may also underlie failure of SC regeneration. This was demonstrated in a limb-girdle muscular dystrophy patient with missense mutation in POGLUT1 (protein O-glucosyltransferase 1), in whom a decrease in Notch signaling was associated with muscle degeneration and loss of SCs (Servián-Morilla et al., 2016).

Intrinsic as well as extrinsic defects can be caused by the disease’s primary mutation.

In case that the mutation is in protein expressed within SCs, the primary mutation itself can impair SC function as demonstrated in DMD and LGMD2H mouse models (Kudryashova et al., 2012; Dumont et al., 2015). If not critical for SC function, the primary mutation may change structures of the ECM and basal lamina, altering SC niche, which may lead to microenviromental defects and ineffective SC activation (Gattazzo et al., 2014b).

Regeneration defects in more than one stage have been reported in several dystrophy types. However, the origin of these defects was elucidated in later studies, when new and powerful technologies enabled more accurate detection and analysis of the various components of the pathophysiology, beyond the primary mutation.

Until recently, the dystrophin gene, *DMD*, was considered to be expressed in myofibers and not SCs. Accordingly, the main contribution to DMD pathology was assumed to be limited to myofiber membrane fragility due to absence of dystrophin, leading to continuous necrosis with progressive fibrosis and muscle wasting (Morgan and Zammit, 2010). Hence, current therapies mainly focus on preventing dystrophy by targeting the myofiber. However, in a recent study, using RNA-sequencing (RNA-Seq) and microarray techniques, dystrophin expression was detected in a subset of SCs and postulated to have an important role in their polarity and asymmetric divisions. Therefore, a primary intrinsic SC dysfunction contributing to DMD pathogenesis has been suggested (Dumont et al., 2015). Evidences for active and unbalanced proliferation in DMD with no change in SC number compared to healthy muscle are available (Boldrin et al., 2009, 2015). However, this process of proliferation is impaired due to abnormal and uncontrolled SC divisions, impaired self-renewal, and eventual decline in the SC pool with time.

In accordance with these results, a recent study at our laboratory supports an intrinsic SC defect in muscle of the *mdx* mouse model of DMD. This study examined 8-week-old *mdx* mouse whole muscle transcriptome using RNA-Seq and found unsynchronized upregulation of all major regeneration transcription factors: Pax7, Myf5, MyoD, MyoG, and Mamster compared to WT muscle, indicating unbalanced active regeneration and SC uncontrolled activation, which may increase the proportion of abnormal cell divisions (Yanay et al., 2019).

## Congenital Muscular Dystrophy Type 1A

Congenital muscular dystrophy type 1A (LAMA2-CMD), also known as merosin-deficient congenital muscular dystrophy type 1A (MDC1A), is a devastating incurable disease, caused by mutations in *Lama2* gene encoding the α-subunit of laminin-211. Mutations in laminin-211, a key anchor basement membrane protein, disrupt the link between cytoskeleton, basement membrane, and ECM, rendering the muscle fiber sarcolemma fragile.

Laminin 211 interacts with α-dystroglycan, a member of the dystrophin–glycoprotein complex (DGC; Spence et al., 2002). This complex links ECM to the actin cytoskeleton of the myofiber, thereby protecting skeletal muscle membrane against contraction-induced damage (Petrof et al., 1993).

Independently, laminin-211 interacts with β1 integrin, a part of the α7β1 integrin complex that gathers a large number of proteins and, like the DGC, functions as a structural link between ECM and actin cytoskeleton, hence similarly playing a significant role in protecting skeletal muscle against contraction-induced injury. Recent studies suggest that the DGC and integrin complex have compensatory abilities in maintaining sarcolemma integrity, as demonstrated by much more severe muscle pathology in double-mutant knockout mice for dystrophin and α7β1 integrin than mice lacking either dystrophin or α7β1 individually (Allikian et al., 2004; Guo et al., 2006; Rooney et al., 2006).

In LAMA2-CMD, either partial or complete absence of laminin-211 expression leads to increased muscle vulnerability to injury with ensuing severe clinical features in children, as well as in LAMA2-CMD mouse models.

Several useful mouse models of LAMA2-CMD are available, demonstrating correlation between laminin-211 expression and disease severity, thus representing heterogeneity in their clinical presentation. Very severe forms of muscular dystrophy are represented by *dy^W^/dy^W^* and *dy^3k^/dy^3k^* models. These models have a similar severe dystrophic phenotype, with life spans of only a few weeks of age, commensurate with almost absent or completely absent laminin-211 expression in *dy^W^/dy^W^* and *dy^3k^/dy^3k^* models, respectively. Moderate severity with reduced life span of 6 months of age represents the *dy/dy* mouse, which has reduced laminin-211 expression and primary mutation still unknown. Mild muscular dystrophy represents the *dy^2J^/dy^2J^* mouse model, with spontaneous mutation in the LN domain resulting in partial deficiency, hind limb paralysis by 3–4 weeks of age, and decreased life span compared to wild type (Xu et al., 1994; Sunada et al., 1995; Miyagoe et al., 1997; Gawlik and Durbeej, 2011; Durbeej, 2015).

## Impaired Regeneration in LAMA2-CMD

Previous studies have provided some clues that the proliferation stage in the regeneration process is impaired in LAMA2-CMD.

One clue for impaired regeneration in LAMA2-CMD, suggested by Kuang et al. (1999) is that a major contributor to muscle disease is abortive regeneration in the *dyW* mouse model. They reported immature myofibers and excessive mononuclear cell death in this model.

Next, in 2005, Girgenrath et al. (2005) showed in the same *dyW* model that mononucleated cells, and in particular muscle SC population, were decreased compared to WT and *mdx* mice, and therefore an altered proliferation stage was assumed. Thus, they hypothesize that SC poor proliferation may be one of the mechanisms underlying the lack of successful regeneration in LAMA2-CMD muscle (Girgenrath et al., 2005).

Only a few studies exploring LAMA2-CMD transcriptome and proteome have been published (Taniguchi et al., 2006; van Lunteren et al., 2006; Moreira Soares Oliveira et al., 2018). As for human data, muscle transcriptome was studied in a single LAMA2-CMD patient at the age of 8 months, using microarray technique with costume chip representing a limited number of 5,600 genes expressed in muscle. Most of the upregulated genes in this patients’ muscle were ECM components, which according to the author reflect active fibrosis and poor muscle regeneration (Taniguchi et al., 2006).

In mouse models, a single study demonstrating gene expression profiling in *dy/dy* using microarray technique found a limited number of genes differentially expressed in the diaphragm (van Lunteren et al., 2006). Genes with altered expression in the diaphragm belonged to cell motility, development, immune response, cellular adhesion, and collagen synthesis. In addition, a study by de Oliveira et al. (2014) described proteomic analysis in the *dy^3k^/dy^3k^* mouse model, showing approximately 100 differentially expressed proteins compared to WT, mainly involving metabolic processes, calcium binding, or ECM protein expression (fibrosis) in diaphragm and gastrocnemius muscles (de Oliveira et al., 2014).

Correlation between clinical phenotype and gene expression was mainly suggested for fibrosis in studies of LAMA2-CMD; thus, therapies so far have largely focused on amelioration or prevention of fibrosis (Nevo et al., 2010; Elbaz et al., 2012; Yu et al., 2013; Accorsi et al., 2016).

In our recent article, next-generation sequencing *via* RNA-Seq technique was applied in the *dy^2J^/dy^2J^* mouse (Yanay et al., 2019). Using this method, we detected a large number of novel significantly differentially expressed genes in quadriceps muscle of 8-week-old *dy^2J^/dy^2J^* mice.

The most significant finding was downregulation of three key myogenic stem cell factor genes: Pax3, Pax7, and Mamstr (Figure 1B). This demonstrated an abnormal regeneration process, which mainly points to impaired SC self-renewal in the *dy^2J^/dy^2J^* mouse model compared to WT.

These results are in agreement with ours and others histological results and previous clinical observation demonstrating poor proliferation and high degree of fibrosis in LAMA2-CMD patients and mouse models (Girgenrath et al., 2005; Yanay et al., 2019).

Earlier studies demonstrate that with absence of Pax3/Pax7 expression, cells undergo apoptosis or adopt alternative nonmuscle lineages (Soleimani et al., 2012), possibly explaining the mechanism underlining very early tissue replacement by fibrosis and fat in LAMA2-CMD models. Transient activation of Pax3 expression in cultures of primary myoblasts results in enhanced proliferation in these cells (Conboy and Rando, 2002; Kuang et al., 2006).

Not only the environment is important for SC functionality, but SCs may also have an impact on their own environment, as loss of SCs may also increase muscle fibrosis. Thus, the presence of normal SCs is required to maintain functional niches that support regeneration (Morrison and Spradling, 2008).

SC involvement in additional myopathies pathologies was reported for XL myotubular myopathy, SEPN1-related myopathies, and as a primary cause for EMARDD (early-onset myopathy with areflexia, respiratory distress, and dysphagia; Castets et al., 2011; Logan et al., 2011; Boyden et al., 2012; Lawlor et al., 2012; Di Gioia et al., 2017). Mutated genes expressed in myotubes as well as in SCs may alter myofiber maturation during embryogenesis, and early growth is postulated to be the cause for the early onset of pathology and weakness in these disorders. Similarly to these congenital myopathies, there are also indications for laminin-211 expression in LAMA2-CMD SCs (Schuler and Sorokin, 1995; Vachon et al., 1996; Morgan and Zammit, 2010). In addition to its roles in prevention of myofiber injury, laminin-211 has therefore been suggested to have a role in myoblast proliferation and SC differentiation and regulation (Girgenrath et al., 2005; Morgan and Zammit, 2010). SC dysfunction in LAMA2-CMD due to an intrinsic defect or extrinsic/microenvironment defects, or a combination of the two, should be further studied.

Understanding SC contribution to LAMA2-CMD muscle pathology may suggest new therapeutic strategies, as current therapies mainly target myofiber damage rather than the SC regeneration process.

## Signaling Pathways Underlying Muscle Regeneration

**Figure 2:**
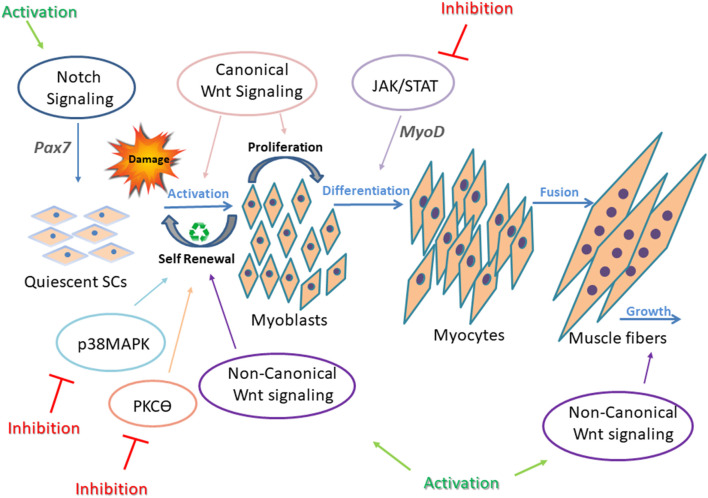
Underlying mechanisms in impaired regeneration during myogenesis. Main regeneration signaling pathways in muscular dystrophy and the corresponding targets for intervention are presented. In adult healthy skeletal muscle, all signaling pathways are tightly controlled and highly synchronized for proper SC differentiation. This is well demonstrated by the interplay between Notch and Wnt signaling. Upregulation of Notch maintains SCs in a quiescent state and promotes self-renewal of activated SCs through upregulation of Pax7. Canonical Wnt signaling antagonizes the effects of Notch signaling, thus allowing the progression through differentiation. Also, canonical and non-canonical Wnt signaling oppose each other, as canonical Wnt signaling regulates the differentiation of muscle SCs, and noncanonical Wnt signals (through ligand Wnt7a) mediate self-renewal (symmetric divisions), migration of SCs, and growth of muscle fibers. p38MAPK pathway regulates toward asymmetric division and SC self-renewal. In dystrophic muscle, signaling is altered and unsynchronized, resulting in defective regeneration. Continuous intervention to activate or inhibit abnormal signaling in SCs failed to improve muscle regeneration. Thus, a dynamic/cyclic regulation of signaling is necessary to balance regeneration of SCs in order to improve long-term regenerative defects of dystrophic muscles (Jiang et al., 2014; Tierney et al., 2014). Cyclic administration of Wnt7a, Notch activators, and JAK/STAT, MAPK, and PKCθ inhibitors have shown beneficial effects on the regeneration process in dystrophic muscles.

Multiple signaling pathways are involved in skeletal muscle regeneration, and each of them is tightly controlled and regulated to enable efficient muscle repair. Notch, Wnt, Janus kinase/signal transducers and activators of transcription (JAK/STAT), Mitogen-activated protein kinase (MAPK), Nuclear factor kappa-light-chain-enhancer of activated B cells (NF-κB), and Transforming growth factor beta (TGF-β) signaling pathways, and others have been intensely studied, and their contribution to developmental and adult muscle regeneration confirmed (Brack et al., 2007; Bjornson et al., 2012; Parker et al., 2012; von Maltzahn et al., 2012a; Tierney et al., 2014). Each signaling pathway is critical for accurate regeneration, but above all, their synchronization and balanced cross-talk are most important for proper regeneration (Figure 2). For instance, in adult skeletal muscle, SCs express high levels of Notch to remain in a quiescent state and prevent Pax7 induced differentiation (Conboy and Rando, 2012; Wen et al., 2012; Fujimaki et al., 2018). On the other hand, with upregulation of the canonical Wnt signaling pathway, SC differentiation is activated (von Maltzahn et al., 2012a). Thus, a precise timing to switch from Notch to Wnt signaling is required for proper SC differentiation (Brack et al., 2007). While the canonical Wnt signaling drives differentiation of SCs, mainly through the ligand Wnt3a, noncanonical Wnt signaling is responsible for mediating self-renewal and migration of SCs, and also growth of muscle fibers through Wnt7a ligand (von Maltzahn et al., 2012a).

Examples for noncanonical Wnt signaling pathways include PCP (planar cell polarity), Wnt/Ca^2+^, and PI3K/AKT/mTOR signaling cascades. Interplay between these factors upon Wnt signaling results in activation of small GTPases, Rac, and Rho, leading to cytoskeletal remodeling, are essential for myoblast fusion.

Additional signaling pathways involved in SC regeneration include p38MAPK signaling, which is highly regulated to permit asymmetric division and SC self-renewal (Troy et al., 2012).

Also, JAK/STAT signaling pathway activates myogenic differentiation by regulating expression of specific genes such as MyoD (Wang et al., 2008).

## Impaired Satellite Cell Regeneration Signaling Pathways in Muscular Dystrophy

Unsynchronized SC signaling pathways resulting in defective muscle regeneration has been described in several muscular dystrophies.

Decrease number of SCs in dystrophic muscle has been associated with a reduced Notch signaling pathway (Jiang et al., 2014; Servián-Morilla et al., 2016).

Activation of Notch signaling was shown to improve self-renewal capacity of SCs in *mdx* mice (Jiang et al., 2014) and ameliorate DMD phenotype in Golden Retriever muscular dystrophy dogs (GRMD; Vieira et al., 2015).

Recently, Fiore et al. (2020) suggested that lack or pharmacological inhibition of protein kinase C theta (PKCΘ), which modulates several signaling pathways in muscle, leads to increased Notch signaling and improved muscle repair and SC self-renewal ability in *mdx* mice. Muscles from limb-girdle muscular dystrophy patients show decreased Notch signaling and a dramatic reduction in SC pool (Servián-Morilla et al., 2016). Significant rescue of the myogenesis was demonstrated in these patients by increasing Notch signaling (Servián-Morilla et al., 2016).

Canonical Wnt signaling dysregulation has been reported in multiple muscle pathologies, such as DMD (Trensz et al., 2010), FSHD (Block et al., 2013), and OPMD (Abu-Baker et al., 2013).

Muscle lacking secreted factor, Wnt7a, a Wnt signaling ligand, exhibited a marked decrease in SC number following regeneration, whereas Wnt7a overexpression enhanced muscle regeneration and increased SC numbers. Wnt7a also induced myotube hypertrophy and a shift in fiber type toward slow-twitch in human primary myotubes (von Maltzahn et al., 2012b). Intramuscular treatment with Wnt7a increased activated SC number, myofiber size, and muscle force of *mdx* mice.

Inhibition of dysregulated p38MAPK signaling pathway in *mdx* mice improved SC self-renewal and mice phenotype (Smythe and Forwood, 2012; Wissing et al., 2014).

In addition, pharmacological inhibition of JAK/STAT signaling (by Calbiochem) increased numbers of SCs, enhanced muscle repair, and enhanced functional performance in aged healthy mice (Price et al., 2014).

Both canonical Wnt and TGF-β2 signaling are chronically elevated in *mdx* mouse muscle tissue (Biressi et al., 2014).

NF-κB and TGF-β signaling were overexpressed and involved in the pathophysiology of the *dy^2J^/dy^2J^* mouse (Elbaz et al., 2012, 2015). Also, it is well known that chronic activation of NF-κB signaling contributes to DMD pathology, promotes necrosis and inflammation, and inhibits muscle regeneration (Proto et al., 2015). NF-κB inhibition improves *mdx* dystrophic muscle regeneration by directly promoting lineage progression of muscle progenitor cells and by increasing progenitor cell survival (Proto et al., 2015).

Moreover, using canonical pathway analysis by Ingenuity Pathway Analysis, RhoA, NF-κB, epithelial-mesenchymal transition (EMT), TGF-β, and PKCθ were found to be altered in the LAMA2-CMD mouse model examined by RNA-Seq method (Yanay et al., 2019).

## Potential Therapies to Enhance Regeneration

**Figure 3:**
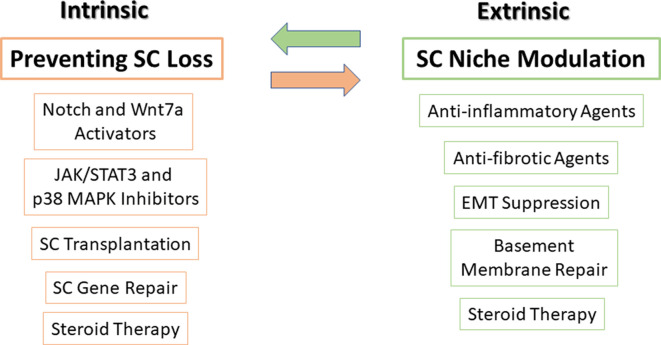
Potential therapies to target SCs and their niche. Very tightly controlled interactions exist between SCs and their microenvironment niche, thus, in order to optimize treatment, targeting both SCs (intrinsic factors) along with their microenvironment niche (extrinsic factors) should be carried out simultaneously. On the left side, SC transplantation, modulation of major signaling pathways in SCs, mediation of gene delivery and repair, and steroid treatment can prevent SC loss and improve their function. On the right side, using anti-inflammatory and antifibrotic agents, suppression of epithelial-mesenchymal transition (EMT), basement membrane protein repair, and steroid treatment can restore SC niche. Steroid treatment is presented in both sides as it affects both SCs and satellite niche.

As previously mentioned, a fine-tuned balance between intrinsic signaling pathways and extrinsic factors is required to correctly control SC function. We therefore subdivide potential therapies to enhance regeneration in muscular dystrophy into those that affect intrinsic factors, extrinsic factors, or both (Figure 3).

As can be seen in Figure 3, pharmacological inhibitor or activator compounds balance and synchronize cell signaling of different intrinsic factors and may prevent SC loss and improve SC function.

Preliminary evidence for the use of SC transplantation as a potential therapeutic treatment was provided from the results of a phase II clinical trial in OPMD patients (www.clinicaltrials.gov NCT00773227; Périé et al., 2014). In that study, local autologous myoblast transplantation demonstrated significant improvement in patients’ swallowing abilities with no adverse side effects. Because this procedure was accepted as generally safe with good tolerance, it has been extended to a larger cohort of OPMD patients. However, many obstacles to SC transplantation still exist, such as SCs’ limited number, delivery methods, cell source (auto or allogeneic) as reviewed by Almeida et al. (2016), and need to be addressed before considering SC therapy for LAMA2-CMD patients or other muscular dystrophies with more widespread muscle involvement.

SC impaired genes can be targeted using viral vectors. Restoration of dystrophin expression using adeno-associated virus was shown to restore dystrophin in myotubes and restore dystrophin at low levels in SCs in the *mdx* mouse model (Tabebordbar et al., 2016). In addition, using lentiviral vector delivery of microdystrophin to neonatal SCs of *mdx* mice resulted in preservation for 2 years of dystrophin expression in myofibers, demonstrating stable transduction of SCs (Kimura et al., 2009).

Challenges in muscle formation may also be due to extrinsic factors in the microenvironment of SCs.

Host SC niche modulation should also be considered in order to produce efficient SC transplantation. Excess fibrosis and altered ECM composition alters SC functionality, resulting in impaired regeneration (Ross et al., 2012). A functional niche will provide an adjusted microenvironment for donor cells to be received by the hosts’ tissue (Boldrin et al., 2012). Thus, preservation of the defective components of the SC niche is critical in order to optimize stem cell therapies.

Contribution of fibrosis and also chronic inflammation to the pathogenesis of LAMA2-CMD are well documented (Girgenrath et al., 2005; Taniguchi et al., 2006; Elbaz et al., 2012, 2015; Yanay et al., 2019). Improved regeneration can be achieved using anti-inflammatory and antifibrotic agents as demonstrated over the years (Dadush et al., 2010; Nevo et al., 2011; Elbaz et al., 2012; Accorsi et al., 2016). Immunosuppressant cyclosporin A treatment maintained SC number in Col6a1^−/−^ mice following multiple bouts of induced injury (Gattazzo et al., 2014a).

Our recent RNA-Seq results support the significant role not only, as expected, of fibrosis, but also of the immune system contribution to LAMA2-CMD pathogenesis. Our findings also proposed that epithelial–mesenchymal transition (EMT) contributes to the dystrophic tissue fibrosis (Yanay et al., 2019). Thus, development of therapeutic interventions, either anti-inflammatory therapies at disease onset or suppression of EMT, may decrease fibrosis in muscular dystrophy in the mouse model.

Repair and preservation of the basement membrane could also support SC niche and functionality and restore muscle regeneration. Laminin-411 was found to be overexpressed in LAMA2-CMD as an attempt to compensate for the abnormal laminin-211 protein (Patton et al., 1997; Ringelmann et al., 1999). Laminin-411 upregulation was also confirmed in our RNA-Seq of *dy^2J^/dy^2J^*. However, laminin-411 lacks binding domains to laminin-211 receptors. In the study by Reinhard et al. (2017), laminin-411 was used as a scaffold protein to link mini-agrin and αLNNd (a chimeric protein that contains laminin-211 binding parts to a basement membrane component nidogen-1) in order to stabilize the muscle basement membrane. Transgenic expression of mini-agrin composed of laminin-211 binding sites to α-dystroglycan and αLNNd, together with an increased laminin-411 level, restored basement membrane stability, muscle function and size, and animal survival in the *dy^W^/dy^W^* mouse model.

Steroid therapy is the gold standard treatment in DMD, and its effect is associated with elevated regeneration and reduced numbers of inflammatory cells (Sklar and Brown, 1991; Hussein et al., 2006).

Prednisolone treatment was found to upregulate the Mamstr gene in dystrophin-deficient mouse muscle compared to untreated controls (Chadwick et al., 2016) and upregulate Myh and dystrophin in human DMD primary culture (Sklar and Brown, 1991). In a clinical report by Hussein et al. (2010), 6 months’ prednisone therapy was associated with ultrastructural changes in dystrophic muscle and increased numbers of SCs, together with a decreased number of immune system cells (dendritic cells) and fibroblasts in DMD and BMD patients’ biopsies.

## Summary

Recent findings point toward SC involvement in muscular dystrophy pathology.

The pathophysiology of LAMA2-CMD includes impaired SC regeneration in addition to muscle cell degeneration due to a fragile cell membrane, as a result of reduced laminin-211.

In support of this, we characterized in a recent study using RNA-Seq technique, a novel molecular signature of the contribution of specific key genes: *Pax3*, *Pax7*, and *Mamstr*, and signaling pathways, to the impaired muscle regeneration process in the *dy^2J^/dy^2J^* mouse model. These results suggest that the muscle phenotype in LAMA2-CMD may be ameliorated by therapies focused on recovering SC number and function.

In addition, preservation of the host SCs’ niche is also required in order to provide an optimal microenvironment to improve SC function or SCs/stem cell transplantation efficacy.

The lack of Pax3 may point to very early, possibly prenatal dysfunction of SCs; thus, early treatment may be considered, before massive fibrosis accumulation and as a consequence establishment of hostile microenvironments, which presents further difficulty to SC-induced regeneration.

Confirmation of this mouse model data with additional LAMA2-CMD human data is required to advance the development of additional new therapies in LAMA2-CMD.

## Author Contributions

NY and YN wrote the manuscript with support of MR.

## Conflict of Interest

The authors declare that the research was conducted in the absence of any commercial or financial relationships that could be construed as a potential conflict of interest.
